# Monthly Summary

**Published:** 1869-10

**Authors:** 


### MONTHLY SUMMARY.

---

*A Novel Tumor.*—The following extraordinary case of "shamming" is from a late number of the *Indian Medical Gazette.* It is reported by Dr. Macleod Cameron, Civil Assistant Surgeon, Monghyr; Emamun, a Mussulman, aged 15, was brought to me on November 20th, by her parents. They stated that upwards of a year before they had observed a small tumor near the angle of the lower jaw on the left side. It continued to increase slowly; native practitioners failed to give relief, and at last despairing of a cure, they had brought her to have it removed by operation. There was a tumor on the left side of the face, rounded, of the size of a tea-cup. The skin slid easily over it, and at its most prominent part was dusky red, and apparently on the point of ulcerating. The tumor was firm, of a bony consistence, and seemed equally connected with both jaws. The lower jaw was fixed, the mouth nearly closed, and the girl complained of great pain. In spite of the suffering she had undergone, she had not lost flesh, and the right cheek was plump and rounded. On separating the lips, to inspect as far as possible the interior of the mouth, I observed the ends of two flat bands of a black color which fell from the tumor into the mouth. On inspecting these somewhat minutely (which was a matter of some difficulty, as she was perpetually starting back, and complaining of great pain) I noticed certain lines which seemed to indicate that the bands were pieces of cloth inserted into a cavity in the tumor, or that cloth of some sort had been recently placed in contact with them, so as to leave its impression. I asked the parents if any cloth had been introduced into the mouth; but they asserted that such was not the case, and the girl corroborated their statement. I now seized the band with forceps, and, using a little force, succeeded in removing it; the girl shrieking loudly and endeavoring to seize my hand. The band was simply a piece of cloth. On examining the mouth I saw what was undoubtedly a second piece of cloth, which I also removed, and thus I went on removing piece after piece till every vestige of the tumor disappeared. The girl looked foolish and sulky. The parents seemed stupefied, and could not at once realize that their daughter's illness was pure deception. They brought her to me again on the following day. There was not the slightest trace of disease. The teeth were sound; the jaws well formed. The right cheek was, as I have said before, plump and round; the left was thin, and hung flaccid and void of expression. The centre of the cheek, which formed the most prominent part of the tumor, was now shrivelled up, like the skin of a withered apple. The tumor was composed of twenty-three pieces of cloth, weighing, when washed and dried, four ounces.—*World*

---

*Biting the Snake to Preserve the Teeth.*—The superstition mentioned in the following item prevails very extensively in some portions of the South and West.

An old lady, in Iowa, complimented on the beauty and preservation of her teeth, ascribed it to having "bitten the snake." She explained that in childhood her father held a rattlesnake by the head and tail, and each of the children bit along the whole length of the backbone, just indenting the skin, as a preventive of tooth-ache and decay, and the old lady believes to the present in the efficacy of such an operation.—*The Medical & Surgical Reporter*.

---

*Styptic Paper.*—A mode for carrying about ferric chloride as a ready styptic has been invented in Paris, which consists in dipping paper into a decoction of 1 lb. of benzoin, 1 lb. of alum in 4 gallons of water, which has been kept boiling for four hours, with renewal and skimming. The paper is left in the filtered solution for some time until saturated; it is then dried, and painted over with a solution (neutral) of chloride of iron; this is then dried, folded, and wrapped in an impervious cover.—*Druggists Circular.*

---

*Chromic Acid*—Is perhaps the best, and certainly the least painful of all caustics. It is extremely well adapted to destroy all morbid growths or excrescences. Not painful, and not liable to spread like most caustics. It has been successfully used to destroy cancroid excrescences on or near the os uteri.—*Druggists Circular.*

---

*Foreign Objects in the Lungs.*—A metallic tube composed of zinc and copper, one-half an inch long and weighing eleven grains, was recently in a fit of coughing expelled from the lungs of a girl twelve years of age living in New York City. The tube, being the whistle of an india-rubber air ball, was two years ago accidently forced into the upper part of the larynx, and thence, in the attempts to remove it by emetics, was lodged in the lungs. During the whole of the period mentioned, the girl suffered from an oppressive sensation on the chest and from continued coughing. During one of the paroxysms the tube was ejected. This occurrence gives a strong illustration of the remedial force of nature which is sometimes successful in affording relief when all the resources of surgery and medicine have been tried and failed.—*Med. & Surg. Reporter.*

---

*Sweet Quinine.*—W. Proctor, Jr.. the able editor of the *Philadelphia Journal of Pharmacy*, has analyzed the preparation sold under the name of *sweet quinine*, and says it is not quinia but mainly the alkaloid *cinchonia.* precipitated from the sulphate of cinchonia, dried and triturated with an impure glycyrrhizin prepared from liquorice root. Cinchonia, he says, "however tasteless, is not quinine, nor does its commercial value approach that of quinine so closely as it is made to do in the garb of 'sweet quinine'. When physicians want cinchonia they can get it by prescription, and it is not in accordance with our ideas of fair dealing to serve it up as a new substance."—*Pacific Med. and Surg. Journal.*

*Mercury Fumes.*—The dangerous action of mercury upon the health of laborers in looking-glass manufactories is well known. According to a recent investigation, however, it is shown that it is a mercurial dust rather than the sublimed vapor that produces disease. This dust can be prevented from flying by adding one-half per cent of sodium to the mercury, which completely neutralizes any such tendency. The saving of the mercury more than equals the cost of the sodium.—*Med. & Surg. Reporter.*

---

*Strange Montrosity.*—We have received the following singular account from a gentleman in New York :

" A correspondent of the *Dantzic Gazette* writes as follows from Dirschau : 'Last Sunday, February 1, at Schliewen, near Dirschau, a young and blooming shepherd's wife was delivered of a girl otherwise sound, but having on the lower part of her back (*auf unterm Ruckentheile*) a tumor as big as two good sized fists. In this tumor, which is covered by the skin, is a very lively fœtus, whose well-developed mass may be felt through the walls of the tumor. Its limbs indicate a growth of from five to six months. The father called in the health commissioner, Dr. Preuss from Dirschau, and begged him to remove the tumor together with the fœtus. The Doctor, however, after he had long and carefully examined it, declared that there was a possibility in this extraordinary case, of the child in the tumor (whose existence and active motions were palpable to all present) coming to fruition. No physician could be justified in destroying this marvellous being. Rather it ought to be protected and cherished. The new-born girl is of unusual strength and beauty, and takes the breast very cheerfully.' "

---

*Position of the body Favoring Sleep.*—Dr. Kenney, in the *Dublin Quarterly Journal*, gives some curious facts on this subject. He says : I had read in some book that sleep was often prevented from the position of the person not being in the right direction, and that to insure the soundest sleep the head should lie to the North ; and, strange as this idea may at first appear, it has more in it than might be supposed. He then gives several instances in which this idea was carried out with satisfactory results in obstinate wakefulness, where narcotics had failed to induce sleep.

Man is such a bundle of nerves and notions that there is no telling what striking results may sometimes follow even a change of position in persons of highly nervous and susceptible temperament.—*St. Louis Med. Reporter.*

---

*Muscular Contraction After Congelation.*—Dr. Richardson performed a somewhat remarkable experiment in confirmation of an observation originally made by John Hunter. Hunter observed that if fresh muscles be frozen, in the act of thawing muscular contraction will take place. Dr. Richardson has devised an experiment by which the fact may be audibly and visibly demonstrated. If a freshly denuded muscle be first frozen with ether spray and then immersed in a freezing mixture, it may be afterwards gradually thawed by being held horizontally by a piece of cord over water at 120°. If one end of the cord be made fast in the vessel holding the water and the other end be fastened to the trigger of a pistol, and the temperature of the muscle be then suddenly raised by placing a spirit lamp under the vessel containing the water, and heating it to 125°, the muscles contract sufficiently quickly and forcibly to fire off the pistol.—*London Med. Times & Gaz.*

---

*Cure for Toothache.*—The *Lancet* says toothache can be cured by one drachm of Calvert's carbolic acid. A gelatinous mass is precipitated, a small portion of which, inserted in the cavity of an aching tooth, invariably gives immediate relief.

---

*Use of Gun-Cotton in Dentistry.*—This statement appears absurd at first, as if dentists used gun-cotton as an explosive agent; but the fact is, that quite recently the collodion mode of gun-cotton, hardened by evaporation. as a varnish, into thin sheets, has been used as a substitute for the objectionable vulcanized rubber, as a basis for support of false sets of teeth. For this purpose different sheets are softened by ether, and pressed together in the mould, which is made in a way similar to that in use for making the platinum or India-rubber sets.

One of the objections to the vulcanized rubber sets of teeth is the dark color, which can only be corrected by vermillion, which gives it a reddish color, somewhat similar to that of the gums.

Vermillion, however, being a compound of mercury, seriously affects the health of some persons, whose peculiar constitution renders them very sensitive to the influences of this pernicious metal.

In drying, collodion contracts considerably, and the only additional trouble, in making objects of dry collodion, is to make the moulds larger, by repeated casting and recastings in Plaster; the plaster expanding every time a little, the last mould obtained may be sufficiently enlarged to compensate for the shrinkage of the material. Sets of teeth made on collodion are much lighter and stronger than on any other material thus far employed for that purpose, and, no doubt, will soon come into general use in the United States, as the dentists of this country are among the most progressive in the world.—*Druggists Circular.*

---

*New Hæmostatic Collodion.*—Dr Carlo Panesi (*Arch. Med. Belges*) has prepared a collodion similar to that of Richardson;

| | |
|---|---|
| Officinal collodion.................................. | 100 parts. |
| Carbolic acid........................................ | 10 " |
| Pure tannin.......................................... | 5 " |
| Benzoic acid......................................... | 3 " |

To be agitated until the mixture is perfect.

This collodion has a brown color. Submitted to evaporation it leaves as residue a pellicle similar to that of the ordinary collodion. It adheres strongly to the tissues to which it is applied and coagulates the blood instantly.—(*Union Med. de la Gironde*) —*Med. Record.*

---

*Change of Chemical Nomenclature.*—Instead of sulphate of copper we shall now say copper sulphate, or cupric sulphate; instead of sulphate of iron, ferrous sulphate; instead of per-sulphate of iron, ferric sulphate. Bicarbonate of soda is monosodic carbonate, or hydrogen and sodium carbonate or hydrosodic carbonate Sesqui-carbonate of soda is dihydro-tetrasodic carbonate. See *Fownes' Chemistry,* the latest authority. In another decade or so, we shall have another revolution in nomenclature. And why not? Do not the fashions of ladies' bonnets change?.—*Pacific Med. and Surg. Journal.*

*Prussic Acid.*—We learn from the *Journal des Connaissances Medicales* that, at the last sitting of the French Academy of Medicine, Dr. Scouttetenn communicated the substance of an essay which created quite a sensation. It was a posthumous disquisition on hydrocyanic acid, found among the papers of the late celebrated Professor Schonbein, of Baden. The question discussed was, whether there was a test for the above-mentioned liquid besides those of M. Liebig and M, Buignet, which, within certain limits, may reveal the presence of prussic acid, but are insufficient to fix its quantity and detect a crime with certainty. Professor Schonbein then proceeds to describe a reagent discovered by himself, and delicate enough to bring out to view even the millionth part of a drop, whether diluted with water, or vaporized in the air; a circumstance affording new proof of the incalculable divisibility of matter. Dr. Scouttetten, who lives at Metz, announced, in his communication, that he had repeated the late Professor Schonbein's, experiments, with the aid of two chemists, MM. Guebin and Pont, and that he begged to submit some of the test-paper prepared by himself to the Academy for further trial. The specimen forwarded was of the kind called filtering paper, and had been soaked in a solution cf three gms. of guaiacum resin in 100 gms. of alcohol. To use it, a solution of ten decigr, of sulphate of copper in fifty gms. of distilled water should be made, and the paper which is white, cut into narrow slips. One of the latter being wetted with the solution, it is then exposed to the action of the minute quantity of hydrocyanic acid dtssolved in water, and suspended in the air: the paper will then instantly turn blue. Dr. Scouttettenn remarks that these slips of paper will be useful in examiuing the quality of the medicinal waters or syrups containing a very small quantity of the acid. The paper need only be placed on the unstoppered neck of the phial containing the medicine, and the blue color will at once become visible. Various other experiments are described, all tending to the same result.—*Med. & Surg. Reporter.*

---

*Sulpho-Vinic Acid.*—*Vitriol* mixed with alchol, is used in London as a summer drink. Of course you do not take the liquors in their original strength; you stir one part of the acid by measure, together with three parts of rum, gin, brandy, or whiskey, and put a small teaspoonful of the mixture in a tumbler of cold water, flavored with a dash of lemon-raspberry or some other syrup. It is said to be good for the stomach, and does not increase perspiration as do some drinks containing vegetable acids. It also has a tonic action on the vascular system. Continental *Cafes* use this mixture in concocting their *liqueurs*; but it would be much safer for the teeth and stomachs of the drinkers to use *Phosphoric* acid in place of the Sulphuric.—*Med. Gazette.*

---

*Remedy for Carious Tneth.*—Nitric ether and sulphate of alumina are mixed so as to form a paste, which is applied to the cavity. It never occasions any inconvenience, the most violent tooth-ache is promptly relieved, and, after several applicatlons, the affected tooth becomes insensible.—*Sweitzer. Wochenschr.*—*Am. Jour. of Pharmacy.*

---

